# *Atp6ap2* ablation in adult mice impairs viability through multiple organ deficiencies

**DOI:** 10.1038/s41598-017-08845-7

**Published:** 2017-08-29

**Authors:** Olivia Wendling, Marie-France Champy, Solène Jaubert, Guillaume Pavlovic, Aline Dubos, Loic Lindner, Hugues Jacobs, Manuel Mark, Roy Combe, Isabelle Goncalves Da Cruz, Hervé Luche, John S. Mudgett, Thomas Rosahl, Tania Sorg, Marie Malissen, Patrick T. Reilly, Yann Hérault

**Affiliations:** 1 CELPHEDIA-PHENOMIN, Institut Clinique de la Souris (ICS), CNRS, INSERM, University of Strasbourg, 1 rue Laurent Fries, F-67404 Illkirch-Graffenstaden, France; 2 Centre d’Immunophénomique, CIPHE, PHENOMIN, INSERM US012, CNRS UMS3367, UM2 Aix-Marseille Université, 13288 Marseille, France; 3 Institut de Génétique et de Biologie Moléculaire et Cellulaire, Illkirch, France; 4 Centre National de la Recherche Scientifique, UMR7104 Illkirch, France; 5 Institut National de la Santé et de la Recherche Médicale, U964 Illkirch, France; 6 Université de Strasbourg, Illkirch, France; 7 Merck Research Laboratories, 2000 Galloping Hill Rd, Kenilworth, New Jersey USA 07033

## Abstract

*ATP6AP2* codes for the (pro)renin receptor and is an essential component of vacuolar H+ ATPase. Activating (pro)renin for conversion of Angiotensinogen to Angiotensin makes ATP6AP2 attractive for drug intervention. Tissue-specific ATP6AP2 inactivation in mouse suggested a strong impact on various organs. Consistent with this, we found that embryonic ablation of *Atp6ap2* resulted in both male hemizygous lethality and female haploinsufficiency. Next, we examined the phenotype of an induced inactivation in the adult animal, most akin to detect potential effect of functional interference of ATP6AP2 through drug therapy. Induced ablation of *Atp6ap2*, even without equal efficiency in all tissues (aorta, brain and kidney), resulted in rapid lethality marked by weight loss, changes in nutritional as well as blood parameters, leukocyte depletion, and bone marrow hypoplasia. Upon *Atp6ap2* ablation, the colon demonstrated a rapid disruption of crypt morphology, aberrant proliferation, cell-death activation, as well as generation of microadenomas. Consequently, disruption of ATP6AP2 is extremely poorly tolerated in the adult, and severely affects various organ systems demonstrating that ATP6AP2 is an essential gene implicated in basic cellular mechanisms and necessary for multiple organ function. Accordingly, any potential drug targeting of this gene product must be strictly assessed for safety.

## Introduction

ATP6AP2 (ATPase H(+)-transporting lysosomal-interacting protein 2, also known as renin/prorenin receptor, ER-localized type I transmembrane adaptor, and V-ATPase M8.9 subunit) is a single transmembrane-domain containing protein that is required for vertebrate development^1, 2^.

It is a promiscuous protein-interaction partner for an array of factors of varied functions. At the cell membrane, it interacts with renin or prorenin^2–4^. The complex formation of ATP6AP2 with Renin/Prorenin affects the activity of both proteins. For Renin/Prorenin, the interaction with ATP6AP2 enhances proteolytic activity toward Angiotensin II (AngII), whereas for ATP6AP2, the interaction causes activation of intracellular signaling pathways resulting in secretion of inflammatory and fibrotic factors^5^. Importantly, the interaction of a secreted extracellular domain, resulting from proteolysis of ATP6AP2 in the golgi, is able also to bind to renin thus allowing modulation of AngII without intracellular signaling. The physiological relevance of this interaction is supported by evidence from animal models^6, 7^.

ATP6AP2 also functions as a critical associated factor in vacuolar H+ ATPase, a membrane transporter critical for maintaining low lysosomal pH and, thereby, sequestration/destruction of cellular waste. Defects and inhibition of vacuolar H+ ATPase (V-ATPase) results in perturbed autophagy in various systems^8, 9^ indicating that the deficient autophagy reported upon ATP6AP2 loss-of-function^10, 11^ is likely due to reduced vacuolar H+ ATPase activity. Indeed, the lack of viability of *Atp6ap2*-deficient mouse embryonic stem (ES) cells and organisms before heart development suggest that its role in vacuolar H+ ATPase activity is essential for development^1, 12^. Additionally, ATP6AP2 was identified as a necessary gene for canonical WNT signaling in Drosophila and Xenopus, due to impaired cellular acidification^13–15^.

The involvement of ATP6AP2 in human pathologies is diverse, suggesting a critical function in various organ systems. Consistent with its role in renin signaling, ATP6AP2 polymorphisms have been linked to clinical hypertension^16, 17^. It has also been implicated in regulating insulin secretion in the pancreas, suggesting a role in diabetes mellitus^18^. In the central nervous system, mutations of ATP6AP2 have been found in multiple families with hereditary X-linked CNS disorders including parkinsonism^19–21^. Recently, a mutation in an alternate V-ATPase subunit, *ATP6AP1*, was reported to be associated with immune abnormalities, hepatopathy, and cognitive impairment^22^.

In light of these implications in human disease, ATP6AP2 has been examined as a potential target for therapeutic intervention, including mouse models for loss of ATP6AP2 function. *Atp6ap2*-mutant ES cells fail to generate chimeric mice^1^ making the constitutive gene ablation in mouse impossible. To date, several conditional knockout (cKO) mouse models have been characterized, all with severe phenotypes: nephrotic syndrome and lethal renal failure after podocyte deletion^23^, lethal heart failure after cardiomyocyte deletion^24^, cognitive impairment and neurodegeneration when deleted in glutamatergic neurons^25^, renal hypodysplasia and tubular acidosis after PRR deletion in the ureteric bud^26^, abnormal T-cell, retina, and adipose development after T-cell, photoreceptor, and adipocyte deletion^27–29^ respectively. Induced ablations of *Atp6ap2* in the mouse kidney have been reported reaching 65–80% reduction of *Atp6ap2* transcripts^11, 30^, with symptoms of renal malfunction^11, 30, 31^.

In order to better mimic the effects of potential ATP6AP2 antagonists, we utilized temporally-controlled deletion in the whole mouse as well as in specific tissues. Here we report that *Atp6ap2* loss-of-function in the embryo leads to lethality, both in the hemizygous male as well as in the heterozygous female. In the adult mouse, induced ablation of *Atp6ap2* results in rapid lethality marked by weight loss, changes in nutritional as well as clinical chemistry parameters, leukocyte depletion, and bone marrow hypoplasia. Reconstitution of bone marrow demonstrated that depletion of haematopoietic stem cells (HSC) was cell-autonomous due to loss of ATP6AP2. Extended study in the colon demonstrated that, in addition to disruption of normal crypt morphology, loss of ATP6AP2 led to generation of microadenomas concurrent with loss of the intestinal stem cell marker, Lgr5. Our study suggest that disruption of ATP6AP2 is extremely poorly tolerated in the adult, severely affecting various organ systems, resulting in a phenomenon resembling multiple organ failure in humans.

## Materials and Methods

### Mouse lines

The X-linked *Atp6ap2* conditional knock-out mouse line (*Atp6ap2* ^*cKO*^) has been described previously^25^. Progeny mice were maintained on C57BL/6NTac background. We used the Tg(*CMV-cre*)1Ipc allele, denoted here as CMV-Cre^32^, as a general constitutive Cre driver to remove the selection cassette in initial viability experiments. The Tg(*ROSA26-creERT2*) allele, denoted here as ROSA-CreERT2, was described in ref. 33 and the Tg(*Villin-creERT2*) allele, denoted here as Villin-CreERT2, was described in ref. 34. After tamoxifen or control induction *Atp6ap2* ^*cKO/Y*^ Tg(*ROSA26-creERT2*) animals are termed *Atp6ap2* ^*RosaTAM*^ and *Atp6ap2* ^*RosaVEH*^, respectively. After tamoxifen or control induction *Atp6ap2* ^*cKO/Y*^ Tg(*Villin-creERT2*) animals are termed *Atp6ap2* ^*vilTAM*^ and *Atp6ap2* ^*vilVEH*^, respectively.

### Genotyping

Mutant mice were identified performing PCR on tail genomic DNA. Primers forward, 5′-AGCACTCTCTTCCAGGTATGTTGTG-3′; reverse, 5′-CTGGATCCCGGAGCATGGGTAAAGG-3′ will generate a 280 bp band on the wild type allele (*Atp6ap2* ^*wt*^), a 330 bp band on conditional (*Atp6ap2* ^*cKO*^) allele. Primers forward, 5′-CAGGTGTGCTGCTATTAATAGG-3′; reverse, 5′-CATCTGCACGAGACTAGTGAGACG-3′ will detect a 514 bp band on the *Atp6ap2* ^*cKO*^ allele. Primers forward, 5′-AGCACTCTCTTCCAGGTATGTTGTG-3′; reverse, 5′-GCCCCTCTCTTACAGTTCTATCAGT-3′ will detect a 3528 bp, a 1640 bp, a 326 bp and a 1480 bp band on *Atp6ap2* ^*cKO*^ *, Atp6ap2* ^*Δex2*^ and *Atp6ap2* ^*wt*^, respectively.

### Animals

The mice were maintained in a room with controlled temperature (21–22 °C) under a 12–12 light-dark cycle (light cycle from 7 a.m. to 7 p.m.) with ad libitum access to the food and water. All mice were 14-weeks-old and were fed standard rodent chow D04 (UAR, France) during analysis. Each mouse was injected intraperitoneally (i.p) with either 0.1 mL of 10 mg/kg Tamoxifen (TAM; #T5648, Sigma-Aldrich, St. Louis, U.S.A.) or with vehicle (VEH) alone for 5 consecutive days. Daily measurements of body temperature, food consumption, water intake, and body weight were performed throughout the study.

### Total RNA extraction and first strand cDNA synthesis

Upon euthanasia, the aortas, brains, colons, femurs, ilea, kidneys, livers and white adipose tissues were snap-frozen in liquid nitrogen for expression analysis. After a Trizol® (Life Technologies GmbH, Darmstadt, Germany) separation to improve protein exclusion, total RNA was isolated using the Total RNA isolation Kit (Macherey-Nagel, Hoerdt, France) following the manufacturer’s instructions. RNA concentration and purity was determined by A260 and A280 measurements using a NanoDrop® ND-1000 spectrophotometer (Thermo Scientific, Waltham, U.S.A.). The mean value of A260/A280 ratio for all RNA samples was 2.04 ± 0.3, reflecting high purity and protein absence. RNA integrity was evaluated using a LabChip® 90 system from Caliper Life Sciences (Hopkinton, U.S.A.). An RNA quality score was calculated using Caliper Life Sciences algorithms. To guarantee the quality necessary for expression analysis all samples used in this study presented a RNA quality score value above 6 as advised in ref. 35.

One microgram total RNA was reverse-transcribed using the QuantiTec® Reverse Transcription kit (Qiagen, Hilden, Germany) according to the manufacture’s recommendations. All cDNA samples were diluted 1:5 with DNase- and RNase- free H2O and stored at −20 °C.

### DNA extraction

DNA samples were extracted as described previously^36^. DNA concentration and purity was determined by A260 and A280 measurements using a NanoDrop® ND-1000 spectrophotometer (Thermo Scientific, Waltham, U.S.A.). The mean value of A260/A280 ratio for all RNA samples was 1.86 ± 0.2, reflecting high purity and protein absence.

### Real-time quantitative PCR with SYBR green

qRT-PCR and qPCR were conducted using the LightCycler® 480 detection system, based on LightCycler® 480 SYBR Green I master kit (Roche, Basel, Switzerland). The PCR reaction mixture contained 2 μl cDNA (unknown concentration) or DNA (25 ng/µl), 0.085 μl (0.7 μM) of each primer, 6 μl LightCycler® 480 SYBR Green I master mix and PCR-grade H2O up to a total volume of 12 μl. After initial enzyme activation (one cycle at 95 °C for 10 min), 45 cycles amplification (95 °C for 10 s, 60 °C for 10 s and 72 °C 5 s) were performed in 384-well optical reaction plates (Roche, Basel, Switzerland). To verify that the used primer pair produced only a single product, a dissociation protocol was added after thermocycling, determining dissociation of the PCR products from 65 to 95 °C by increasing 2.5 °C per second. In all negative control samples no amplification signal was detected. Following recommendations^37^, a standard curve was realized to check efficiency of qPCR designs. Relative quantities were thus corrected for efficiency of amplification.

For qPCR analyses, a normalization assay was done for each sample to correct estimated DNA concentration. No knock-out heterozygote samples were used as control as the knock-out heterozygote females seem not to be viable.

### Clinical chemistry

Blood was collected on 4 hours fasted mice 3 days after the last Tamoxifen injection by retro-orbital puncture on isoflurane-anesthetized mice. Blood chemistry was performed on an OLYMPUS AU-400 automated laboratory work station (Beckman Coulter, Brea, U.S.A) using commercial reagents (Beckmann Coulter Biomedicals, Lismeehan, Ireland). The following parameters were measured: glucose, urea, creatinine, Na, K, Cl, total proteins, albumin, calcium, phosphorus, triglycerides, total cholesterol, HDL and LDL cholesterol, triglycerides, LDH, ALAT and ALP. A complete blood count was performed on an Advia 120 Vet (Siemens Healthcare Diagnostic, Saint-Denis, France).

### Immunohistochemistry and TUNEL assays

The proliferation marker Ki67 (Novocastra Laboratories, Newcastle-upon-Tyne, U.K.) was detected by immunohistochemistry according to standard techniques. The rabbit anti-ATP6ap2 (Sigma HPA003156) was validated previously^25^. Briefly, microwave antigen retrieval was performed in 10 mM sodium citrate buffer (pH6.0) and the rabbit anti-ATP6ap2 or the rabbit anti-Ki67 antisera was diluted at 1/500 in PBS containing 0.05% Tween-20. Then sections were blocked with 10% normal horse serum and 0.3% triton X-100. For ATP6ap2 immunostaining, detection of primary antibody was achieved by incubating sections during 1 hour at room temperature with Alexa Fluor 555- conjugated secondary antibody (1:500, Invitrogen Molecular Probes). The sections were mounted with Mowiol 4–88 mounting medium (Citifluor) containing DAPI (5 μg/ml). For Ki67 immunostaining, the signal was amplified using the ABC method (Vector Laboratories, Burlingame, U.S.A.) and peroxidase activity was revealed using AEC (3-amino-9-ethylcarbazole; Vector Laboratories, Burlingame, U.S.A.). TUNEL assays were performed on formalin-fixed paraffin-embedded histological sections using the ApopTag® Plus Peroxidase *In situ* Apoptosis Detection kit (EMD Millipore, Molsheim, France) according to the manufacturer’s instructions. The time of proteinase K treatment was 10 min and peroxidase was detected using AEC (Vector Laboratories, Burlingame, U.S.A.). The sections were counterstained with Harris hematoxylin for Ki67 and TUNEL stainings. The images were acquired using a Hamamatsu Nanozoomer 2.0 (Hamamatsu, Hamamatsu city, Japan).

### Bone marrow smears

The entire femora was dissected and cleaned of all muscles using a dry paper towel. Each femur was cut open along its length with a pair of scissors and the marrow was collected with a fine brush slightly humidified with PBS. A bone marrow smear was obtained by placing the brush on one end of a glass microscope slide, moving it to the other end of the slide with gentle pressure and repeating the process 4 to 5 times. The slides were air-dried during 1 hour at least and stained with May-Grünwald-Giemsa (Merck, Darmstadt, Germany).

### Bone Marrow Chimera

7–8-week-old male B6 SJL (CD45.1) mice were lethally irradiated with one dose of 6 Gy, and injected i.v. with 4.10^6^ bone marrow (BM) cells obtained from femur of either WT B6 (CD45.2) or *Atp6ap2* ^*RosaTAM*^ (CD45.2) mice or a mixture of 2.10^6^ WT B6 SJL (CD45.1)/2.10^6^ *Atp6ap2* ^*RosaTAM*^ (CD45.2). Chimeras were kept throughout the experiment on antibiotic- containing water (0.2% Bactrim, Roche, Germany). 8 weeks after reconstitution, the level of blood chimerism was determined by FACS. At that time, >99% of blood B cells were of donor origin.

### Flow Cytometry

Antibodies used are listed in Suppl. Table S1. Before staining, cells were pre-incubated 10 min on ice with the 2.4G2 antibody to block Fc receptors. In all experiment, Sytox Blue (Invitrogen) was used to exclude dead cells from the analysis. Multiparameter FACS acquisition was performed on a LSRII SORP system (BD Biosciences). Analysis was performed using FACSDiva 8.01 (BD Biosciences) software. Doublets were systematically excluded based on side scatter (SSC) and forward scatter (FSC) parameters.

### Statistical analysis

All the data are expressed as mean+/− SEM. Statistical analysis were performed using two-tailed Student’s t-test for parametric data and Mann-Whitney U-test for non-parametric data. Statistical significances are denoted according to: *p < 0. 05, ** p < 0.01, ***p < 0.001.

### Ethics Statement

All experiments were carried out in accordance with the European Communities Council Directive of 24 November 1986. YH, as the principal investigator in this study, was granted the accreditation 67–369 to perform the reported experiments and the experimental procedures were authorized by the French Ministry of Research committee C2EA-17 under the two protocols N°00127 (June 21st, 2013) and N° 2015071516186528 (with the new numbering system, September 18, 2015).

## Results

### Ablation of the ATP6AP2 function impairs viability

To generate an embryonic deficiency in *Atp6ap2*, the homozygous conditional *Atp6ap2* (*Atp6ap2* ^*cKO/cKO*^) mouse^25^ was bred to a CMV-Cre deletor strain^32^, which could generate knock-out hemizygote males and knock-out heterozygote females. No pups that carried the deleted exon 2 (*Atp6ap2* ^*Δex2*^) allele were obtained showing that the heterozygote knock-out females and hemizygote knock-out males are not viable (Suppl. Table S2). Our result suggest that as observed during embryonic development^1^, complete loss or even loss of a single functional allele of the *Atp6ap2* gene is sufficient to induce mortality.

### Adult ablation of Atp6ap2 with generalized deletion induced rapid mortality

**Figure 1:**
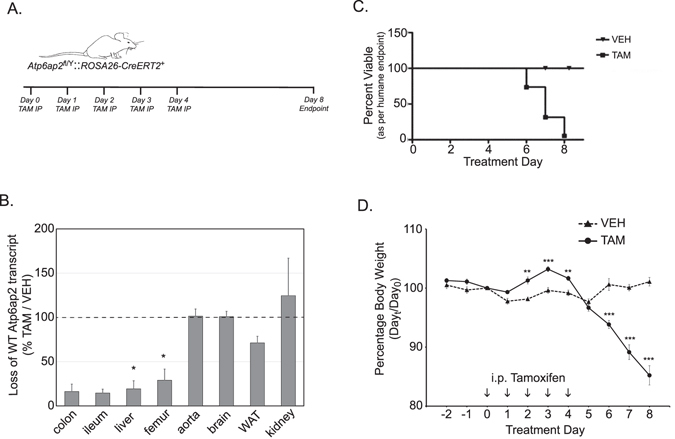
Adult loss of ATP6AP2 compromises viability. (**A**) Initial experiment provided for 5 days of tamoxifen injection with an endpoint at day 8 after first injection in male conditional mice. (**B**) Tissue samples at day 8 were examined for *Atp6AP2* transcript and expressed as a comparative value of tamoxifen treated over vehicle controls [colon and ileum N = 3; femur and liver N = 6; aorta and brain N = 4(TAM) & 2(VEH); kidney and WAT N = 3(TAM) & 2(VEH)]. (**C**) Kaplan-Meyer survival curve of tamoxifen treated animals through the course of this study. Survival is defined as the absence of humane endpoints described in Materials and Methods [N = 23(TAM) & 11(VEH)]. (**D**) Body weight chart of tamoxifen-treated and control mice expressed as a percentage change from day 0 for individual mice [N = 11(TAM) & 9(VEH)]. *TAM*, tamoxifen-treated; *VEH*, vehicle-treated control; *IP*, intraperitoneal.

Since *Atp6ap2* is X-linked, male mice were exclusively examined in subsequent studies for ease of interpretation of a single recombinase-induced gene-inactivation event, whereas female mice may display mosaicism that could confound interpretation. To further study the function of ATP6AP2 in adult mice, we bred the conditional allele with an inducible Cre recombinase expressing strain to generate the bitransgenic Tg(*ROSA26-creERT2* ^+^) *Atp6ap2* ^*cKO/Y*^. The *ROSA26-creERT2* strain was chosen as a generalized deleter as it drives Cre-mediated recombination in a wide range of tissues in controlled manner upon Tamoxifen (TAM) administration^33, 38, 39^. Tam treatment proceeded in a cohort of male mice with daily injections over 5 days (Fig. 1A). Because *ROSA26-creERT2* mediated recombination is mosaic in peripheral tissues and to confirm exon excision, loss of wild-type (wt) *Atp6ap2* mRNA from the floxed allele (cKO) was evaluated in a large panel of organs (aorta, brain, colon, femur, ileum, kidney, liver and white adipose tissue). Organs were chosen based on the expression profile of *Atp6ap2* ^40, 41^ and tissues with known susceptibility to loss of *Atp6ap2* ^23–26^.

The effects of TAM treatment in *Atp6ap2* ^*RosaTAM*^ males on wild type *Atp6ap2* mRNA is shown in Fig. 1B. Loss of *Atp6ap2* transcript was not homogenous. Whereas intestine, liver, and femur demonstrated high levels of loss, the aorta, brain, kidney and white adipose tissue did not respond to TAM treatment. A similar profile of deletion was evident at the DNA level (Suppl. Fig. S1), suggesting more efficient deletion in individual cells expressing greater amounts of *Atp6ap2* mRNA. These results are consistent with the success of recombination driven by the *ROSA26-creERT2* deleter in adult observed in similar driver strain where recombination increases in the heart and kidney increases in the weeks after TAM delivery^38^.

In total, 34 *Atp6ap2* ^*Rosa*^ male mice were injected intraperitoneally either with 1 mg of TAM for 5 days (*Atp6ap2* ^*RosaTAM*^; 23/34) or with oil solvent (*Atp6ap2* ^*RosaVEH*^; 11/34) and were planned for sacrifice 4 days after the last injection. Figure 1C shows the survival curve to humane endpoint for the mice. 16 of the *Atp6ap2* ^*RosaTAM*^ mice reached humane endpoint before day 8. In contrast, the 11 control mice survived without evident adverse effects. *Atp6ap2* ^*RosaTAM*^ mice also displayed a dramatic decrease in body weight from days 4 to 8 (Fig. 1C). Upon sacrifice, the mean body weight of *Atp6ap2* ^*RosaTAM*^ mice was decreased by 15% compared to the *Atp6ap2* ^*RosaVEH*^ mice (22.67 g versus 26.02 g at day 8; Student’s t-test p = 3 × 10^−4^) even with the exclusion of *Atp6ap2* ^*RosaTAM*^ mice that were sacrificed due to humane endpoints. Notably, blood pressure of the control and treated animals remained similar and stable through post-treatment survival (data not shown) although this may reflect an absence of ablation in the heart, as was seen in the aorta. We conclude that continuous *Atp6ap2* gene expression is essential for well-being even when inactivated in a limited number of tissues.

### Adult ablation of *Atp6ap2* disrupts nutritional state and induces hypercholesterolemia

**Figure 2:**
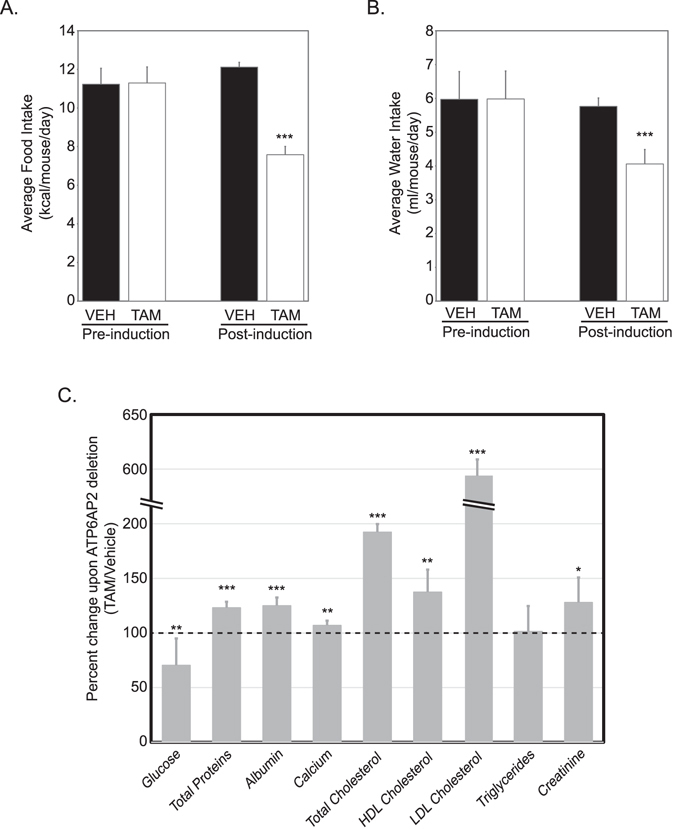
Nutritional defects in *Atp6ap2-*ablated mice. Comparison of (**A**) food and (**B**) water intake in a cohort of animals prior to or after the 5-day injection regimen with vehicle control (filled columns) or tamoxifen (empty columns). Statistical significance is determined by contrasting pre- and post- treatment behaviour of same animals by paired Student’s t-test. [N = 11(TAM) & 9(VEH)]. (**C**) Differences in blood clinical chemistry parameters expressed as a percentage of tamoxifen-treated over vehicle controls [N = 7(TAM) & 9(VEH)]. Statistical significance is determined by contrasting tamoxifen-treated to vehicle control by unpaired Student’s t-test. *p < 0.05; **p < 0.01, ***p < 0.001; *TAM*, tamoxifen-treated; *VEH*, vehicle-treated controls.

By measuring food and water intake prior to (days -2 to 0) and after (days 5–7) the course of TAM treatment we were able to quantify how induced loss of ATP6AP2 affected the nutritional state of these mice. *Atp6ap2* ^*RosaTAM*^ exhibited a marked decrease in food (Fig. 2A) and water intake (Fig. 2B), which was not evident in the vehicle-treated controls.

At day 8, terminal blood draws were analyzed for clinical chemistry parameters including nutrients. Several alterations were evident between *Atp6ap2* ^*RosaVEH*^ and *Atp6ap2* ^*RosaTAM*^ mice (Fig. 2C). Notably, blood glucose levels were significantly decreased in the *Atp6ap2* ^*RosaTAM*^ mice. In addition, total proteins, albumin, creatinine, and calcium levels are significantly higher in the *Atp6ap2* ^*RosaTAM*^ mice than in the controls.

Most striking in this analysis, however, was the evident hypercholesterolemia observed in the *Atp6ap2* ^*RosaTAM*^ mice, with a 41% increase in HDL cholesterol levels and 550% increase in LDL cholesterol levels in the *Atp6ap2* ^*RosaTAM*^ mice. Enzymatic activities, ALP and ALAT, are significantly higher in *Atp6ap2* ^*RosaTAM*^ mice (Suppl. Table S3). The increase in cholesterol levels associated with an increase in hepatic enzyme activities may suggest liver damage. Interestingly, hematoxylin and eosin staining of liver sections showed dying cells in some of the *Atp6ap2* ^*RosaTAM*^ mice (n = 3/6). Alternatively, hypercholesterolemia may be reflect adipose disruption since *Atp6ap2* ^*RosaTAM*^ mice also displayed a significant reduction of the paragenital fat pad (0.06 g ± 0.01 vs. 0.30 g ± 0.03 in the *Atp6ap2* ^*RosaVEH*^ mice; P = 6.8 × 10^−6^).

### Loss of function of *Atp6ap2* leads to hematopoietic defects

**Figure 3:**
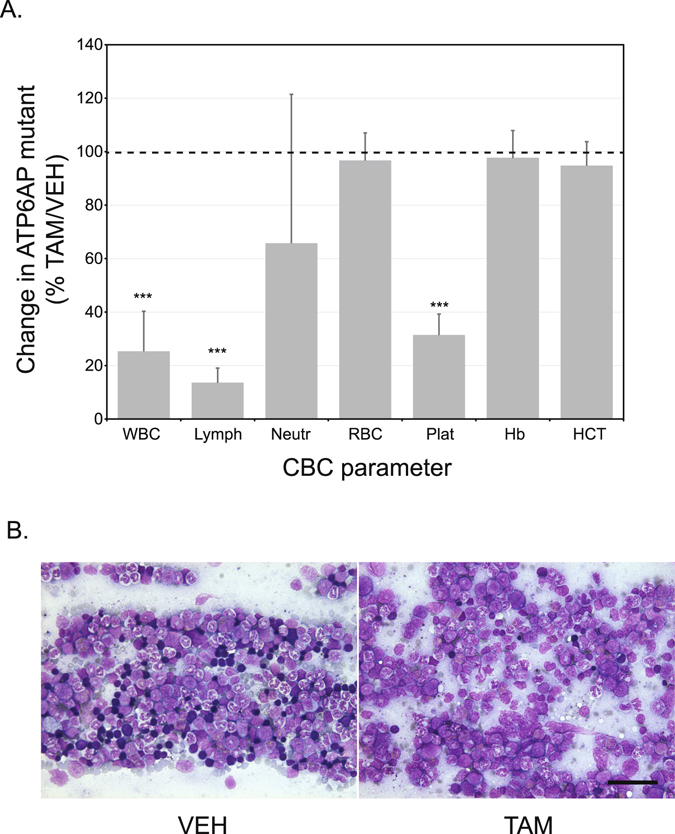
Hematopoietic deficiencies in *Atp6ap2-*ablated mice. (**A**) Clinical blood count parameters are expressed as a percentage of tamoxifen-treated over vehicle controls. [N = 7(TAM) & 8(VEH)]. (**B**) Bone marrow smears stained with May-Grünwald-Giemsa from tamoxifen-treated (TAM) or vehicle controls (VEH). *WBC*, white blood cells; *Lymph*, lymphocytes; *Neutr*, neutrophils; *RBC*, red blood cells; *Plat*, platelets; *Hb*, hemoglobin; *HCT*, hemocrit; ***p < 0.001. The scale bar represents 50 µm.

The terminal blood draw was also used to examine the haematological changes upon loss of ATP6AP2. The complete blood cell count, shown in Fig. 3A, revealed a dramatic decrease in the total number of leukocytes, especially lymphocytes, in *Atp6ap2* ^*RosaTAM*^ mice. The number of platelets was also significantly lower in *Atp6ap2* ^*RosaTAM*^ mice than in the controls. No change was evident in erythrocytes number, hemoglobin and hematocrit levels. In parallel, *Atp6ap2* ^*RosaTAM*^ mice presented lower spleen weight compared to the *Atp6ap2* ^*RosaVEH*^ (0.09 g ± 0.03 versus 0.04 g ± 0.00 absolute value; with p < 0.05). *Atp6ap2* ^*RosaTAM*^ bone marrow smears (Fig. 3b), revealed a near absence of the erythroblastic cell lineage (n = 6/6) as well as a decreased number and dysplasia of the megakaryocytes population. The analysis of cytokines in the blood of *Atp6ap2* ^*RosaTAM*^ mice (Suppl. Table S4) revealed a significant increase of acute inflammatory cytokine TNFα. However, sections of the spleen (n = 6), pancreas (n = 3), heart (n = 3), lung (n = 3), gastrocnemius muscle (n = 3), kidney (n = 3), adrenal glands (n = 3), mesenteric lymph nodes (n = 6), brain (n = 3), brown and white adipose tissues (n = 3) did not show any leukocytic invasion as a sign of inflammation. In addition to the histological bone-marrow defect, we examined other tissues for evidence of aberrant cell death activation. We noted, in particular, that samples from livers demonstrated an increase in apoptotic nuclei (Suppl. Fig. S2). Liver damage may also be noted in clinical chemistry parameters with increased in Alanine amino-transferase and albumin in the blood (Suppl. Table S4). These data suggest that peripheral tissue damage attempts to elicit an inflammatory response through cytokine signaling but inflammation does not ensue due to leukocyte hypoplasia.

### *Atp6ap2*-deficient leukocytes fail to reconstitute immune-deficient animals

**Figure 4:**
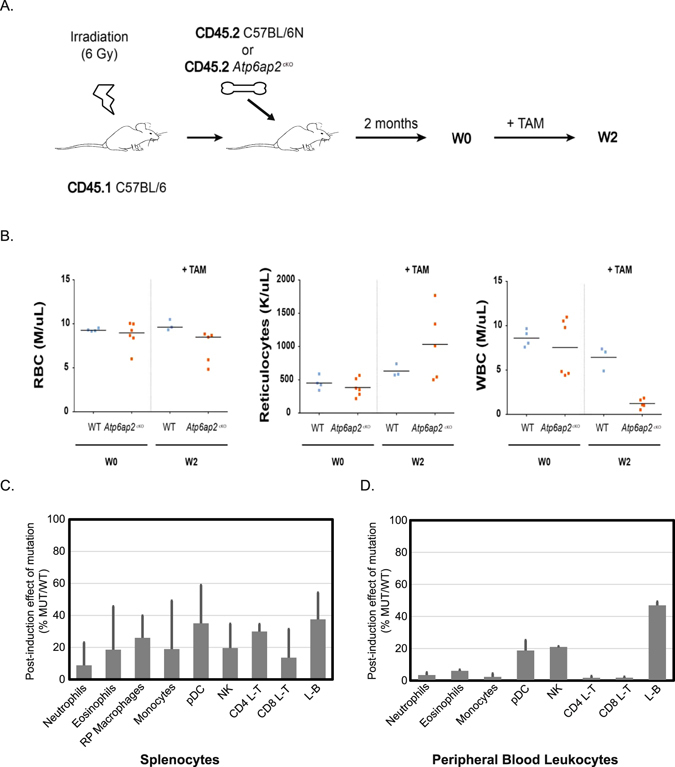
Bone-marrow reconstitution with *Atp6ap2* ^cKO^ does not support full hematopoiesis upon tamoxifen treatment. (**A**) Experimental design for reconstitution of irradiated mice allowing 8 weeks for reconstitution (W0) and two weeks for immune-phenotyping after tamoxifen-treatment regimen (W2). Irradiated SJL CD45.1 host mice are reconstituted with either *Atp4ap2* ^RosaTam^ CD45.2 or WT-CD45.2 BM cells. (**B**) Clinical blood counts from reconstituted *Atp4ap2* ^RosaTam^ or WT-animals at W0 and after tamoxifen treatment (W2). (**C**,**D**) Hematopoietic lineage analysis expressed as a percentage of *Atp6ap2* ^cKO^ over wild-type after tamoxifen treatment for (**C**) spleen [N = 3] or (**D**) blood [N = 3]. *TAM*, tamoxifen; *RP*, red pulp; *pDC*, plasmacytoid dendritic cells; *NKc*, natural killer cells; *Tc*, T lymphocytes; *Bc*, B lymphocytes.

**Figure 5:**
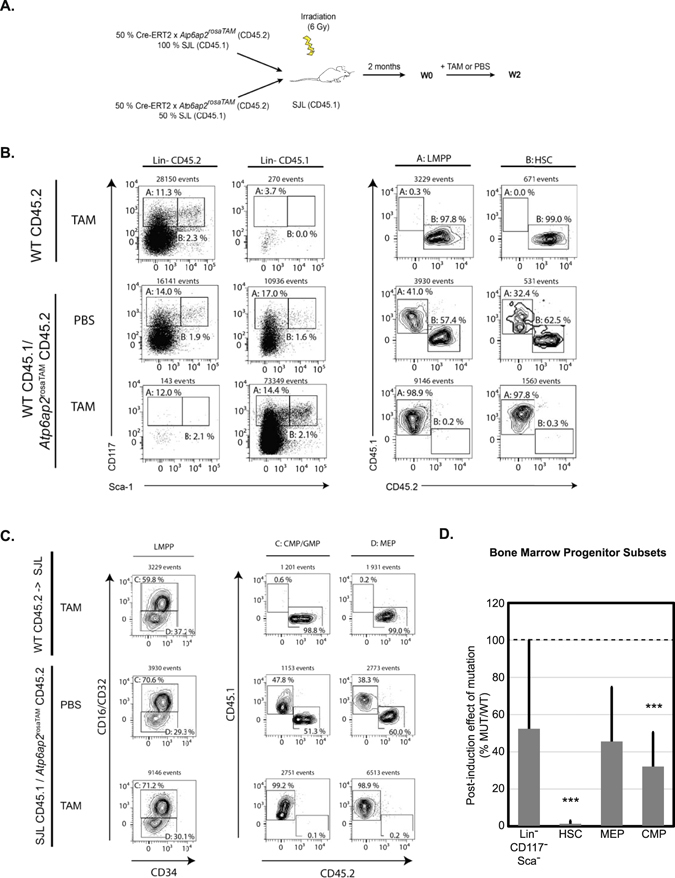
*Atp6ap2* ablation impairs hematopoietic stem cell and progenitor survival in the bone marrow in a cell-intrinsic manner. (**A**) Experimental design of bone marrow chimera reconstitution performed with *Atp6ap2* ^cKO^ mixed BM cells to WT BM cells where each genotype are distinguishable by use of different CD45 congenic markers. (**B**) Bone marrow analysis of hematopoietic progenitor pools in the two classes of reconstituted animals: *MPP* (hematopoietic multipotent progenitor) defined as population I (Lin^−^CD117^+^Sca-1^−^); *CMP/GMP (*common myeloid progenitors/granulocyte-monocyte progenitors) defined as population II (Lin^-^CD117^+^Sca-1^−^CD16/32^+^CD34^hi^), *MEP* (megakaryocyte-erythrocyte progenitors) defined as population III (Lin^−^CD117^+^Sca-1^−^CD16/32^−^CD34^lo^) on the left cytograms. Expression of CD45 congenic markers were used in order to quantify the contribution of *Atp6ap2* ^cKO^ and WT BM cells to the CMP/GMP and MEP progenitor pools after exposure to tamoxifen (TAM) or control treatment (VEH). (**C**) Bone marrow analysis of hematopoietic stem cell pool in the two classes of reconstituted animals. *HSC*, hematopoietic stem cells defined as population IV (Lin^−^CD117^hi^Sca-1^+^). Expression of CD45 congenic markers were used in order to quantify the contribution of *Atp6ap2* ^cKO^ and WT BM cells to the HSC pool after exposure to tamoxifen (TAM) or control treatment (VEH). (**D**) Relative contribution of different genotypes to stem cell and progenitor pools in bone-marrow of reconstituted animals expressed as a percentage arising from *Atp6ap2* ^cKO^ (MUT) reconstituted animals over those of wild-type (WT) [N = 10]; Statistical significances presented are results of Student’s t-test for effect of genotype in the reconstituted animals after TAM treatment.; ***p < 0.001.

In order to assess whether these hematopoietic abnormalities were due to an intrinsic defect of *Atp6ap2* deficiency on the immune system or to an indirect effect due to a peripheral organ failure in *Atp6ap2* ^*RosaTAM*^ treated mice, we generated bone marrow (BM) chimeras (See Fig. 4A). Upon irradiation, the host immune system was depleted and replaced by adoptive transfer of either WT or *Atp6ap2* ^*RosaTAM*^ bone marrow cells. 8 weeks post-transplantation, the contribution of host (CD45.1) and donor (CD45.2) BM on the regeneration of the hematopoietic system was evaluated on peripheral blood leukocytes using congenic markers, respectively CD45.1 and CD45.2. BM cells from both WT and *Atp6ap2* ^*RosaTAM*^ mice could reconstitute equally well myeloid (granulocytes) and lymphoid (L-B and L-T) lineage cells, indicating that untreated *Atp6ap2* ^*RosaTAM*^ BM cells could seed the bone marrow of recipient mice and give rise to all main hematopoietic lineages (Suppl. Fig. S3). Specific deletion of *Atp6ap2* in the immune system was then induced by TAM injection. 2 weeks after treatment (W2), hematological analysis was performed on peripheral blood (Fig. 4B). RBCs were modestly decreased in treated *Atp6ap2* ^*RosaTAM*^ > SJL chimera compared to WT > SJL treated animals. This decrease of overall RBCs was compensated by a 2 times increase of immature reticulocytes number in treated *Atp6ap2* ^*RosaTAM*^ > SJL chimera Wk2 post-treatment. Conversely, peripheral blood leukocytes in treated *Atp6ap2* ^*RosaTAM*^ > SJL chimera were decreased by 80% compared to untreated chimeras, highlighting an intrinsic defect of ATP6AP2 deficiency on the immune system (Fig. 4C). Peripheral blood leukocytes were further characterized by flow cytometry to investigate whether all lineages were equally affected by *Atp6ap2* deletion. As shown in Fig. 4C, all leukocytes lineages were decreased by 80 to 90% in treated *Atp6ap2* ^*RosaTAM*^ > SJL chimera compared to WT > SJL treated animals. Similar profound defect was observed in the spleen of *Atp6ap2* ^*RosaTAM*^ > SJL chimera (Fig. 4D, Suppl. Table S5). In order to investigate whether this relative decrease in hematopoietic cells was due to a central defect of hematopoiesis, competitive bone marrow chimera analysis was performed. WT and *Atp6ap2* ^rosaTAM^ BM cells were mixed to a 1:1 ratio (Fig. 5A). 2 months after adoptive transfer, progenitor pools and HSCs of treated and untreated chimeras were analyzed in BM by flow cytometry. Upon TAM treatment, 98% remaining cells found in the multipotent progenitor pool of mixed BM chimera were of WT origin. Staining using CD34 and CD16/32 markers was then performed to resolve megakaryocyte erythroid progenitor (MEP), common myeloid progenitors (CMP) and granulocyte-macrophage progenitors (GMP) subsets (Fig. 5B). In order to analyze whether HSC pool was impacted to the same extent, additional CD117 and Sca1 staining was performed on lineage negative cells (CD5^−^CD19^−^Ly6G^−^CD11c^−^). Similarly to MEP and CMP/GMP, HSCs were composed of 98% WT cells in treated mixed BM chimeras 2 weeks post TAM treatment (Fig. 5C and D). Therefore, ATP6AP2 deficiency leads to a central and likely cell-autonomous defect in hematopoiesis in HSCs as well as progeny lineages, which is not a secondary effect of peripheral organ damage in the intact *Atp6ap2* ^*RosaTAM*^ mice. A cell-autonomous viability defect is consistent with the expression pattern of *Atp6ap2* in mouse hematopoietic cells as described by the Immunological Genome Project (ImmGen)^42^, wherein *Atp6ap2* is broadly expressed in hematopoietic lineages including all hematopoietic stem cell subsets.

### Intestinal disorganization and microadenomas formation upon loss of ATP6AP2

**Figure 6:**
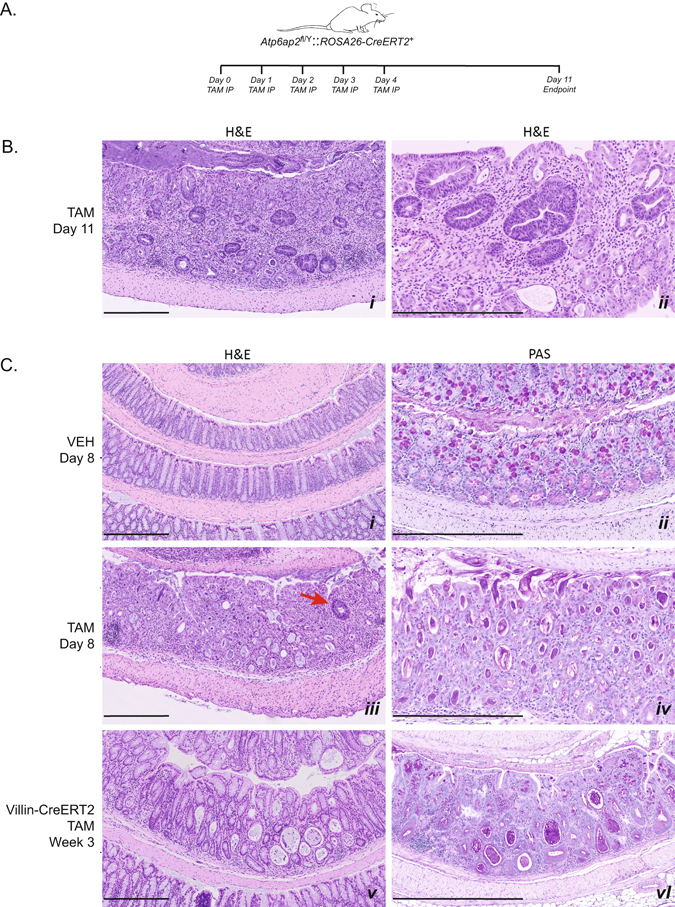
Adult *Atp6ap2* ablation results in colon disorganization and microadenomas. (**A**) Experimental design allowing seven days post-tamoxifen regimen to provide progression of colon defects. (**B**) Histological examination by haematoxylin and eosin (H&E) stain of *Atp6ap2* ^*cKO*^ colons 11 days after beginning tamoxifen regimen. (**C**) H&E and Periodic acid Schiff (PAS) staining of colons of vehicle-control (VEH, panels i and ii), tamoxifen-treated (TAM, panels iii and iv), and *Atp6ap2* ^*vilTAM*^ (panels v and vi). *IP*, intraperitoneal injection. Red arrow indicates early microadenoma and scale bars represent 400 µm.

Due to the evident nutritional defects (Fig. 2) and the presentation of diarrhea in 4 TAM-treated mice surviving to day 7, we closely examined the intestines of *Atp6ap2* ^*RosaTAM*^ mice at necropsy. Macroscopic examination of the inner intestinal surfaces of three *Atp6ap2* ^*RosaTAM*^ mice did not show any apparent defects. Hematoxylin and eosin staining of histological sections of *Atp6ap2* ^*RosaTAM*^ mice revealed a hyperplasia of the colonic mucosa as well as an epithelial dysplasia characterized by highly disorganized colonic glands: instead of tubular crypts and villi, regularly spaced and of constant caliber, the glands of *Atp6ap2* ^*RosaTAM*^ mice appeared convoluted with irregular lumens (Fig. 6C compare panels i and iii). However, no change in the cellular composition of these glands were observed and no nuclear atypia were detected although inflammatory infiltrates were observed in the submucosa of *Atp6ap2* ^*RosaTAM*^ animals (n = 6/6). The glandular abnormalities were further characterized by Periodic acid Schiff staining^43^ showing distended enlarged crypts with mucus accumulation (n = 5/5) (Fig. 6C compare panels ii and iv).

To more closely examine this effect, a second experiment was initiated allowing for 25% weight loss in order to extend our targeted examinations of intestinal function. This experiment provided for harvest at day 11 (Fig. 6A). These endpoint modification allowed extended survival. When we examined *Atp6ap2* ^*RosaTAM*^ mice seven days later after the last TAM injection, additional intestinal abnormalities could be found. Specifically, we found a high density of microadenomas in the colons of *Atp6ap2* ^*RosaTAM*^ mice (Fig. 6B). Upon closer examination of *Atp6ap2* ^*RosaTAM*^ mice from shorter treatment times, we found a microadenoma in a colon (Fig. 6C panel iii). No such aberrations were evident in control animals suggesting that microadenoma formation is a late event, potentially due to disorganization of the colonic epithelium, after loss of ATP6AP2. Although occurring in colons with reduced ATP6AP2 and a disorganized pattern of proliferation, these microadenomas stain positive for ATP6AP2 suggesting that they result from cells which have escaped ATP6AP2 ablation (Suppl. Fig. S4).

To dissociate the general defects obtained with the ROSA-Cre deleter, we generated mice *Atp6ap2* deletion specifically in the intestine using Villin-creERT2 mice *(Atp6ap2* ^*vil*^). The murine Villin promoter provides stable and homogeneous expression of transgenes in small and large intestine along the crypt-villus axis, in differentiated enterocytes, as well as in the immature, undifferentiated cells of the crypt^34^. Four *Atp6ap2* ^*vil*^ were injected with 1 mg of Tamoxifen for 5 days and were sacrificed 12 weeks after the last tamoxifen injection for the *Atp6ap2* ^*vilTAM*^ mice. While less pronounced than seen in the *Atp6ap2* ^*RosaTAM*^ mice, the *Atp6ap2* ^*vilTAM*^ mice also demonstrated disorganized colonic epithelia, suggesting that proper organization of the intestinal epithelia requires ATP6AP2 in this tissue.

### Atp6ap2 deficiency induces intestinal disorder via aberrant proliferation, cell-death, and differentiation pathways

**Figure 7:**
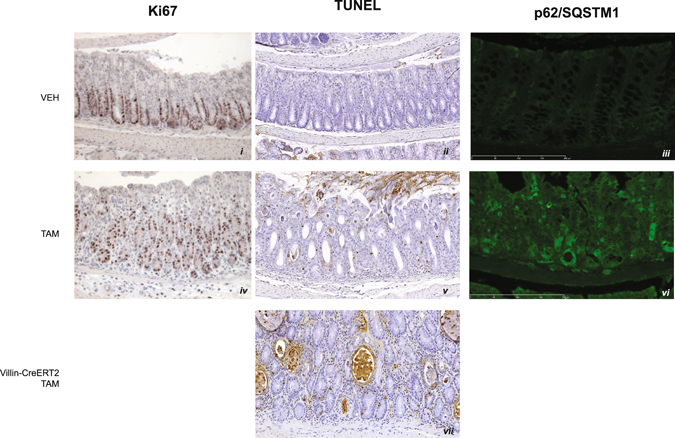
Aberrant cell death pathways in colon epithelia after *Atp6ap2* ablation. Contrasting the physiology of *Atp6ap2* ^*RosaVEH*^ (VEH, panels i–iii), *Atp6ap2* ^*RosaTAM*^ (panels iv–vi), and *Atp6ap2* ^*vilTAM*^ (panel vii) for proliferation marker Ki67 (panels i and iv), apoptosis marker TUNEL (panels ii, v, vii), and a marker of auophagic activity p62/SQSTM1 (panels iii and vi).

To further characterize the abnormalities of the colon, we examined cell proliferation using the Ki67 marker, the TUNEL assay, and P62/SQSTM1 autophagic markers, respectively. In normal colonic epithelium, cell proliferation is restricted to crypt wall such that Ki67-positive cells are not evident on the luminal side of the crypt-villi axis (Fig. 7 panel i). Upon inactivation of *Atp6ap2*, however, Ki67 labelled nuclei were distributed throughout the colonic epithelium including on the luminal side of the colonic crypts (Fig. 7 panel iv; n = 4/5). Intriguingly, the overall amount of proliferation, as denoted by Ki67 staining, was similar with or without ATP6AP2 suggesting that the disorganization is not an effect excessing cell proliferation. Indeed, the disorganized proliferation may be an effect of the disorganized organ structure rather than a cause. Our examinations of bone marrow and liver suggested that cell survival may be defective in the *Atp6ap2* ^*RosaTAM*^ mice. We, therefore, used the TUNEL assay and P62/SQSTM1 autophagic marker to examine differences in apoptosis and autophagy, respectively. We saw a consistent increase in the number of apoptotic cells in the colons of *Atp6ap2* ^*RosaTAM*^ mice compared to the controls (n = 5/5; Fig. 7 panels ii and v). Increased TUNEL staining was also evident in the *Atp6ap2* ^*vilTAM*^ colons, suggesting that this is a tissue-autonomous effect (Fig. 7 panel vii). A more dramatic effect was seen for autophagy, whereby a high proportion of colon epithelial cells of *Atp6ap2* ^*RosaTAM*^ mice stained for P62/SQSTM1 (Fig. 7 panels iii and vi) indicating defective autophagy in these cells. These data indicate that different physiological and survival pathways are disrupted upon loss of ATP6AP2.

**Figure 8:**
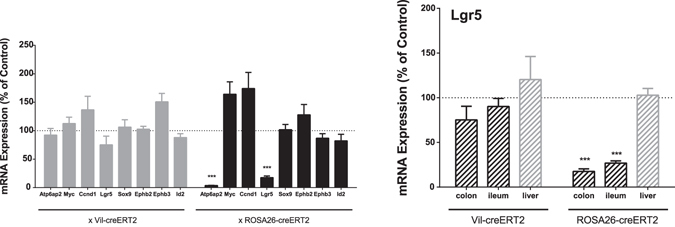
Specific *Lgr5* expression defect in colon upon Atp6ap2 ablation. (**A**) mRNA expression analysis of various intestinal differentiation markers in colonic epithelia of conditional *Atp6ap2* mutation driven by *Villin-CreERT2* or *Rosa26-CreERT2* with or without tamoxifen induction [N = 7]. (**B**) mRNA expression analysis of *Lgr5* in colons, ilea, and livers of conditional *Atp6ap2* mutation driven by *Villin-CreERT2* or *Rosa26-CreERT2* with or without tamoxifen induction [N = 7]. All values are expressed as a percentage of expression in tamoxifen treated colon epithelia over expression in control treated tissues. ***p < 0.001 by Student’s t-test.

The WNT signalling has been shown to be important in adult stem-cell biology and especially in intestinal cell fate determination^44^. Furthermore, ATP6AP2 is directly involved in this pathway^13, 14^. In order to further investigate the impact of ATP6AP2 on intestinal stem-cell biology, we performed expression analysis on colons of *Atp6ap2* ^*RosaTAM*^ mice. Examining a panel of genes known to be involved in intestinal differentiation and stem-cell maintenance^45, 46^ in both *Atp6ap2* ^*vilTAM*^ and *Atp6ap2* ^*RosaTAM*^ in the colon (Fig. 8A). We noted that TAM treatment of *Atp6ap2* ^*vil*^ did not result in a significant reduction in *Atp6ap2* mRNA, suggesting that the *Atp6ap2* targeting is inefficient in this model consistent with the weaker intestinal phenotype. In contrast, *Atp6ap2* mRNA is dramatically reduced in the *Atp6ap2* ^*Rosa*^ mice upon TAM treatment. Although most of the stem-cell markers examined did not demonstrate any statistically significant change in expression upon TAM treatment, *Lgr5* expression was strongly reduced upon TAM treatment, specifically in the *Atp6ap2* ^*Rosa*^ mice. This effect was evident in both the ileum and colon but not the liver (Fig. 8B), where *Atp6ap2* is also effectively targeted in the *Atp6ap2* ^*Rosa*^ mice (Fig. 1B). Since Lgr5 is an important regulator of stem cell maintenance in the intestine, these data suggest that the disorganization and general dysfunction of the intestine upon loss of ATP6AP2 may be due to altered Lgr5 signaling causing dysregulated proliferation and increased activation of cell death pathways.

## Discussion

ATP6AP2 has been a proposed target for therapeutic intervention since it was characterized as having an activating role in (pro)renin signalling. Although a number of previous studies have demonstrated a detrimental impact from loss of ATP6AP2 in the development of different organs, our model has more directly asked how the organism is affected by loss of ATP6AP2 function systemically in the adult. While our model is limited by the fact that the ROSA26-CreERT2 driver leads to partial induction of recombination in peripheral tissues, we have found rapid and serious adverse impacts on numerous organ systems including the bone marrow, liver, and intestine. These data indicate that ATP6AP2 is an important factor in many aspects of organismal survival and that prospective targeting of ATP6AP2 for any of its known functions carries significant risks that would have to be properly examined before progressing to clinical studies.

In addition to male mice that were hemizygous lethal for *Atp6ap2*, we also saw haploinsufficiency in the females deleted for *Atp6ap2*. This may be a dosage effect in the cell, suggesting a high degree of sensitivity to loss of *Atp6ap2*, but also may be due to a mosaic effect of X-chromosome inactivation causing loss of *Atp6ap2* in a critical number of cells where the wild-type copy is inactivated. Regardless of the cellular threshold requirement of ATP6AP2, the haploinsufficiency in the females underlines the critical requirement of this gene in development. The critical requirement of ATP6AP2 in development is consistent with prior publications wherein ATP6AP2 has been demonstrated to be essential early in development^1, 12^ probably due to a basic cellular role, such as supporting V-ATPase activity.

Although our model is limited by the fact that the ROSA26-CreERT2 driver leads to partial induction of recombination in peripheral tissues^38^, the systemic, induced deletion of *Atp6ap2* demonstrated effective deletion in selected tissues. We chose to quantitative analysis of RNA and DNA, as this is more easily interpreted than semi-quantitative or qualitative analysis by immunoblotting. The rapid morbidity after CreERT2 induction by tamoxifen cannot ensure an examination of steady-state gene expression, since residual Cre activation as well as mRNA and protein turnover could still be occurring when these experiments reach a humane endpoint. Unfortunately, relevant tissues for examining the Renin-Angiotensin system of blood pressure regulation either did not demonstrate deletion or were not tested by qRT-PCR. Deletion in the aorta, brain, adipose tissue and kidney were not detected to a large degree in this systemic gene ablation model. While it may be ideal to have a complete inducible systemic knockout, such a system does not currently exist and many of the answers regarding ATP6AP2 function in the Renin-Angiotensin system or cell viability, for organs where it is most prominently expressed may depend on further tissue-specific inducible deletions. Since our study was designed to answer the question of whether systemic targeting of ATP6AP2 could pose additional risks in the clinic, we have effectively answered this question by demonstrating significant effects on metabolism, hematopoiesis, and intestinal biology. Although a particular drug may be able to target the Renin-Angiotensin system, the risks are substantial, with dramatic metabolic changes, reduced weight, and cholesterol metabolism being affected, a severe and rapid leukopenia with defect in bone marrow progenitors concurrent with an increase in TNF-a, as well as serious impacts on intestinal function.

In hematopoiesis, WNT signalling is critical for HSC self-renewal^47^ and ATP6AP2-dependent acidification mediates WNT signalling in xenopus development. Given the severe effects we see in HSC quantities after induction of *Atp6ap2* loss-of-function both in the intact mouse and in hematopoietic grafts, it is likely that defective Wnt signalling is responsible, at least in part, for this effect. It is evident that hematopoietic progeny cells are less affected in our study, albeit in the context of leukopenia, than are the HSCs.

The most intriguing effect seen in an organ was found in the colon. Here we found a highly disorganized and dysfunctional organ within days of deletion of ATP6AP2. Interestingly, disruption of epithelial organization was also seen in the tubules of an inducible renal ablation of *Atp6ap2* ^11^. The authors of that study reached a similar conclusion as we have reached, namely that epithelial disorganization is likely due to increased cell toxicity due to loss of ATP6AP2. The role of ATP6AP2 in the colon has not previously been described and appears to be complex. Upon loss of ATP6AP2, the regular crypt pattern in the colon fades, cell proliferation loses its anatomical restricted, and cell death pathways become activated. Significantly, we found evidence of microadenomas that appear to arise in the residual *Atp6ap2*-competent cells. The rapid onset of these dysplastic lesions, without exposure to mutagen or activation of known oncogenic pathways, lends credence to the disputed theory that colon architecture and aberrant crypt foci are important in colon carcinogenesis^48–50^. We believe that the survival deficiency of the *Atp6ap2*-ablated cells provides that microadenomas only arise from the *Atp6ap2*-competent subset of cells. Unfortunately, the deteriorating health after loss of ATP6AP2, did not allow us to examine the progression of these microadenomas and specific targeting of the intestine using the *Villin* promoter did not recapitulate the effects on this organ. The strongly reduced expression of the crypt-stem cell marker *Lgr5* ^51^ upon loss of ATP6AP2 would point to crypt stem cells being the important cell type causing this phenotype. Somehow ATP6AP2 should play a role in maintaining the gut homeostasis, and this imbalance combined with metabolic and hematopoietic dysfunctions. Diarrhea concurrent with severe weight is predicted to be the proximal cause of morbidity and mortality, focusing clear attention on the apparently critical role of *Atp6ap2* in the intestine.

Our data provide evidence that ATP6AP2 may protect cells from both apoptosis in different tissues. Whereas the livers show apoptotic nuclei after inducing ATP6AP2 loss-of-function, the colon shows enhanced apoptosis associated with defective authophagy. Furthermore, hematopoietic stem cells are cleared in bone marrow of reconstituted animals. Taken together, ATP6AP2 is evidently important for cell survival. While understanding pathway(s) are not crucial to assess the value of a gene product as a potential drug target, it is clearly evident that loss of ATP6AP2 product results in widespread cell viability problems, notably in stem cell compartments.

Known human mutations in ATP6AP2 are limited to splice-site defects and these manifest in CNS disorders^19–21^. The reported mutations cause abnormal ratio of expression of certain isoforms. It has been proposed that the CNS defects are due to a particular sensitivity of neurons to defects in autophagy^25^. In contrast, in this study we have sought a critical exon ablation, which will result in a stronger mutation in this gene. The relatively mild nature of the clinical mutations would explain why human patients carrying ATP6AP2 mutations do not demonstrate the existential problems of the intestine, liver, or hematopoietic system that are evident in our mice. Intriguingly, a new study indicates that an alternate subunit of V-ATPase induces immune abnormalities, hepatopathy, and cognitive impairment^22^, suggesting that many of the phenotypes demonstrated upon *Atp6ap2* deletion are due to V-ATPase dysfunction. The Renin-Angiotensin system cannot be excluded as having a role in the cell survival based on our analysis but, taken together with the early embryonic lethality of the null^1, 12^ (Suppl. Table S2), the autophagy defects seen in our and other studies^10, 11^, and dissimilarity with Renin^52^ and Angiotensinogen^53^ knockout phenotypes, lead us to this conclusion.

We have noted that induced adult deletion of ATP6AP2 shares some common manifestations with clinical Multiple Organ Dysfunction Syndrome (MODS, commonly termed “multiple organ failure”). These common manifestations include high levels of certain cytokines as well as simultaneous intestinal and liver dysfunction. The absence of systemic inflammation (possibly due to leukocyte depletion), respiratory distress, or obvious renal dysfunction argues against this as a model for clinical MODS. Indeed, the degenerating organs in our analyses are the same ones where ATP6AP2 is most effectively targeted, namely liver, intestines, and presumably bone marrow, suggesting that ATP6AP2 is a crucial factor in cell physiology and the simultaneous dysfunction of multiple organs reflect the importance of this gene in multiple tissues. A similar phenotype has recently been described for adult deletion of Wilms’ tumor gene 1^54^.

Different peptides of ATP6AP2 provide for separable activities of this gene product^55^. Indeed, a naturally-occurring soluble (pro)renin receptor has been suggested to have activity for (pro)renin signalling^56, 57^, resulting in augmentation of AngII signals. Whereas, the activities are separable, evolution has covalently linked these two activities in the same gene product. Without clear evidence of an absence of any feedback mechanisms, it would be prudent to perform extensive preclinical testing of any drug targeting portions of ATP6AP2.

## Electronic supplementary material

Supplementary Information
