# Supplementary material for: Atp6ap2 ablation in adult mice impairs viability through multiple organ deficiencies
Source: Sci Rep. 2017 Aug 29;7:9618. doi: 10.1038/s41598-017-08845-7 (PMC5575319; doi:10.1038/s41598-017-08845-7)
Supplement: Supplementary file 1 — Supplementary Information [file 41598_2017_8845_MOESM1_ESM.pdf]

Atp6ap2 ablation in adult mice impairs viability through multiple organ deficiencies

Olivia Wendling[1‡], Marie-France Champy[1‡], Solène Jaubert[2‡], Guillaume Pavlovic[1‡], Aline Dubos[1,3,4,5,6], Loic Lindner[1], Hugues Jacobs[1], Manuel Mark[1,3,4,5,6], Roy Combe[1], Isabelle Goncalves Da Cruz[1], Hervé Luche[6], John S. Mudgett[7], Thomas Rosahl[7], Tania Sorg[1], Marie Malissen[2], Patrick T. Reilly[1], and Yann Hérault[1,3,4,5,6*]

Affiliations

[1] CELPHEDIA-PHENOMIN, Institut Clinique de la Souris (ICS), CNRS, INSERM, University of Strasbourg, 1 rue Laurent Fries, F-67404 Illkirch-Graffenstaden, France

[2] Centre d'Immunophénomique, CIPHE, PHENOMIN, INSERM US012, CNRS UMS3367, UM2 Aix-Marseille Université, 13288 Marseille

[3] Institut de Génétique et de Biologie Moléculaire et Cellulaire, Illkirch, France

[4] Centre National de la Recherche Scientifique, UMR7104, Illkirch, France

[5] Institut National de la Santé et de la Recherche Médicale, U964, Illkirch, France

[6] Université de Strasbourg, Illkirch, France

[7] Merck Research Laboratories, 2000 Galloping Hill Rd, Kenilworth, New Jersey, USA 07033

[‡] These authors contributed equally to the research

[*] **Corresponding author:** Yann Hérault, email: herault@igbmc.fr

Running title: Adult ablation of *Atp6ap2* impairs viability

Supplementary Tables

Antibody Conjugate	Clone	Company	Cat. Number
CD5-BV421	53-7.3	BD Bioscience	562739
CD24-BV421	M1/69	Biolegend	101825
Ly6G-BV421	1A8	BD Bioscience	562737
CD4-V500	RM4-5	BD Bioscience	560782
IA/IE BV510	M5/114.15.2	Biolegend	107635
CD19-BV570	6D5	Biolegend	115535
CD150-BV605	TC15-12F12.2	Biolegend	115927
F4/80-BV605	BM8	Biolegend	123133
CD45.1-BV650	A20	BD Bioscience	563010
CD64-BV711	X54-5/7.1	Biolegend	139311
CD11b-BV711	M1/70	BD Bioscience	563168
CD8a-BV786	53-6.7	Biolegend	100749
CD11c-BV785	N418	Biolegend	117335
CD43-FITC	S7	BD Bioscience	553270
Ly6C-FITC	AL-21	BD Bioscience	553104
CD117-PE	2B8	BD Bioscience	553355
Ly6C-PE	AL-21	BD Bioscience	560592
CD11b-PECF594	M1/70	BD Bioscience	562287
CD127-PECF594	SB/199	BD Bioscience	562419
SigF-PECF594	E50-2440	BD Bioscience	562757
CD5-PECy5	53-7.3	BD Bioscience	553024
CD19-PECy5	1D3	eBioscience	15-0193-82
CD43-PECy5	1B11	Biolegend	121216
Ly6G/C PECy5	RB6-8C5	Biolegend	108410
CD3e-PECy55	145-2C11	eBioscience	35-0031-82
CD45R-PECy55	RA3-6B2	eBioscience	35-0452-82
CD317-PECy7	eBio927	eBioscience	25-3172-82
Sca-1-PECy7	E13-161.7	Biolegend	122513
CD135-APC	A2F10.1	BD Bioscience	560718
CD161-APC	PK136	BD Bioscience	550627
CD16/32-A700	93	eBioscience	56-0161-82
MHCII-A700	M5/114.15.2	Biolegend	107622
CD45.2-APCCy7	104	BD Bioscience	560694

Suppl. Table S1. Antibody used in this study. Same antibodies against congenic markers were used in the three panels of this study (Peripheral blood, Spleen and Bone Marrow)

A.

Sire	Dam		Resulting Genotypes						
			Male			Female			Chi Square p-value
			Atp6ap2$^{wt/Y}$	*Atp6ap2*$^{neo/Y}$	*Atp6ap2*$^{\Delta ex2/Y}$	*Atp6ap2*$^{+/+}$	*Atp6ap2*$^{neo/+}$	*Atp6ap2*$^{\Delta ex2/+}$	
Atp6ap2$^{+/Y}$::*CMVCre*$^{+}$	*Atp6ap2* $^{neo/+}$::*CMVCre*$^{-}$	expected	11.25	5.625	5.625	11.25	5.625	5.625	2.8 x 10^{-5}
		obtained	19	3	0	21	2	0	

B.

Sire	Dam		Resulting Genotypes			
			Male	Female		Chi Square p-value
			Atp6ap2$^{wt/Y}$	*Atp6ap2*$^{fl/+}$	*Atp6ap2*$^{\Delta ex2/+}$	
Atp6ap2$^{fl/Y}$::*CMVCre*$^{-}$	*Atp6ap2*$^{+/+}$::*CMVCre*$^{+}$	expected	23.5	11.75	11.75	2.0 x 10^{-4}
		obtained	28	19	0	

Supplementary Table S2. Embryonic lethality from consitutive loss of Atp6ap2 function. Different breedings for Sires and Dams in tables A & B demonstrate (A) hemizygous male lethality and (B) female haploinsufficiency.

		TAM			VEH		
		Mean		SEM	Mean		SEM
**Glucose****	**mmol/L**	10	±	0.51	14.2	±	0.97
**Total cholesterol*****	**mmol/L**	5.00	±	0.12	2.60	±	0.03
**HDL-cholesterol****	**mmol/L**	2.35	±	0.15	1.71	±	0.07
**LDL-cholesterol*****	**mmol/L**	2.56	±	0.10	0.43	±	0.02
**Triglyceride**	**mmol/L**	0.74	±	0.04	0.73	±	0.04
**Creatinine***	**µmol/L**	11.0	±	0.87	8.6	±	0.25
**Total proteins*****	**g/L**	64	±	0.86	52	±	0.73
**Albumin*****	**g/L**	35	±	0.8	28	±	0.4
**Calcium*****	**mmol/L**	2.46	±	0.03	2.23	±	0.02
**Urea**	**µmol/L**	7.7	±	0.3	7.0	±	0.3
**Sodium**	**mmol/L**	154	±	2.0	150	±	1.0
**Potassium**	**mmol/L**	5.5	±	0.1	4.8	±	0.1
**Chloride**	**mmol/L**	115	±	1.0	114	±	1.0
**Phosphorus**	**mmol/L**	2.23	±	0.04	2.18	±	0.1

Suppl. Table S3. Blood analysis on *Atp6ap2*^{cKO} mice treated with (TAM) or without (VEH) tamoxifen. Means ± SEM of plasma chemistry and blood analysis results [N=7(TAM) & 9(VEH)]. The data were compared using unpaired student's t-test. Parameters with statistically significant differences are indicated as *($p<0.05$), **($p<0.01$), ***($p<0.001$). *HDL*, high-density lipoprotein; *LDL*, low-density lipoprotein

		TAM			VEH		
		Mean		SEM	Mean		SEM
**White blood cells*** **	**x10^3/µl**	1.73	±	0.34	6.84	±	0.67
**Neutrophils**	**x10^3/µl**	0.42	±	0.13	0.64	±	0.03
**Lymphocytes*** **	**x10^3/µl**	0.35	±	0.04	2.58	±	0.28
**Red blood cells**	**x10^6/µl**	9.67	±	0.38	10	±	0.09
**Platelets*** **	**x10^3/µl**	343	±	31	1094	±	28
**Hemoglobin**	**g/dl**	13.6	±	0.51	13.9	±	0.16
**Hematocrit**	**%**	45.5	±	1.5	48	±	0.63
**IL-6**	**pg/ml**	27.0	±	11.9	4.9	±	1.7
**TNF-alpha** **	**pg/ml**	20.7	±	5.2	5.2	±	1.0
**IL-1-alpha**	**pg/ml**	22.5	±	8.3	14.27	±	8.5
**IL-1-beta**	**pg/ml**	8.0	±	3.7	20.8	±	7.9
**ALAT** *	**U/L**	85	±	12	41	±	10
**ALP*** **	**U/L**	390	±	24.0	72	±	3.0

Suppl. Table S4. Blood analysis on *Atp6ap2*^{cKO} mice treated with (TAM) or without (VEH) tamoxifen. Means ± SEM of plasma chemistry and blood analysis results [N=7(TAM) & 9(VEH)]. The data were compared using unpaired Student's t-test. Parameters with statistically significant differences are indicated as *(p<0.05), **(p<0.01), ***(p<0.001). *HDL*, high-density lipoprotein; *LDL*, low-density lipoprotein

		Peripheral Blood											
		Control			Mutant			Splenocytes Control			Splenocytes Mutant		
		Mean		SEM	Mean		SEM	Mean		SEM	Mean		SEM
**Neutrophils**	**%**	23.27	±	6.55	0.80	±	0.31	2.011	±	0.064	0.177	±	0.165
**Eosinophils**	**%**	2.21	±	0.25	0.13	±	0.03	0.283	±	0.041	0.053	±	0.043
**Red Pulp Macrophages**	**%**							1.035	±	0.136	0.269	±	0.075
**Dendritic Cells**	**%**	0.043	±	0.014	0.008	±	0.004	0.372	±	0.086	0.130	±	0.042
**NK cells**	**%**	0.85	±	0.09	0.18	±	0.05	1.611	±	0.155	0.316	±	0.138
**CD4+ T cells**	**%**	7.52	±	1.47	0.13	±	0.05	0.458	±	0.040	0.137	±	0.003
**CD8+ T cells**	**%**	5.50	±	1.46	0.10	±	0.02	0.513	±	0.041	0.070	±	0.053
**B cells**	**%**	29.75	±	3.00	13.96	±	3.46	46.43	±	1.26	17.44	±	4.43

Supplementary Table S5: Hematopoietic lineage analysis of peripheral blood [N=3] and spleens [N=3] after tamoxifen treatment. Data also presented graphically in Figure 4.

Supplemental Figure Legends

Supplemental Figure S1: Tissue samples at day 8 were examined for *Atp6ap2* exon2 inclusion in genomic DNA and expressed as a comparative value of tamoxifen treated over vehicle controls. [colon and ileum N=3; femur and liver N=6; aorta and brain N=4(TAM) & 2(VEH); kidney and WAT N=3(TAM) and 2(VEH)]. Error bars represent one standard deviation. *WAT*; white adipose tissue.

Supplemental Figure S2: Abnormal cell morphology of hepatocytes after Atp6ap2 ablation. H&E staining of mouse livers from *Atp6ap2*^{RosaVEH} (left) or *Atp6ap2*^{RosaTAM} (right). Black bar represents 200 μm.

Supplemental Figure S3: Baseline reconstitution of hematopoiesis with *Atp6ap2*$^{Rosa\text{-}CreERT2}$ bone marrow. Spleens were examined for immune cells constituents two month after reconstitution with either wildtype bone marrow (top) or *Atp6ap2*$^{Rosa\text{-}CreERT2}$ bone marrow (bottom) before induction. Representative examination shows little difference in total splenocytes, T cells, B cells, or neutrophils between the different genotypes prior to induction.

Supplemental Figure S4: Immunohistochemical examination of ATP6AP2 in colon. A. Immunohistochemical stainings of colons from ATP6AP2 competent mice stained without (panels *i.* and *ii.*) or with (panels *iii.* and *iv.*) primary anti-ATP6AP2 antisera (Sigma, cat#HPA003156). White bar represents 400 μm (panels *i.* and *iii.*) or 200 μm (panels *ii.* and *iv.*); B. Immunohistochemical staining of colons from ATP6AP2 (panels *i.* and *ii.*) and Ki67 (panels *iii.* and *iv.*) in colons show above-background staining of ATP6AP2 in microadenomas (white markers) in *Atp6ap2*^{RosaTAM} mice. White bar represents 800 μm (panels *i.* and *iii.*) or 400 μm (panels *ii.* and *iv.*).

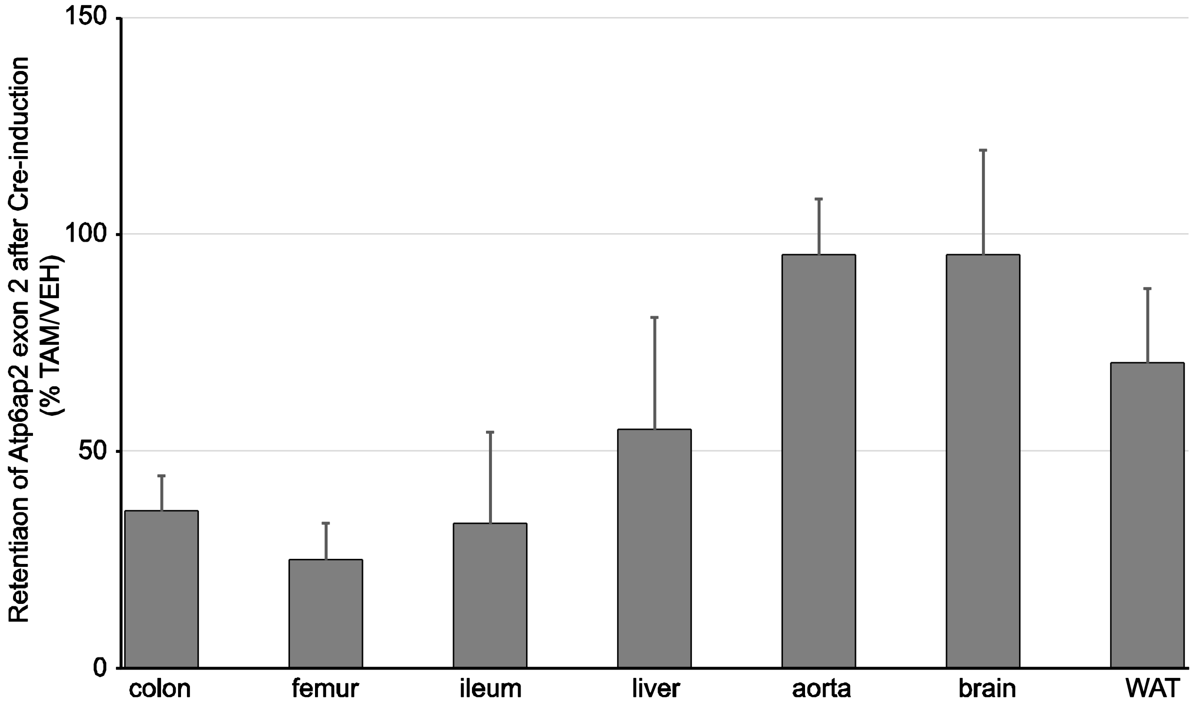
Retentiaon of Atp6ap2 exon 2 after Cre-induction
(% TAM/VEH)
150
100
50
0
colon
femur
ileum
liver
aorta
brain
WAT

Suppl Fig. S1

VEH TAM

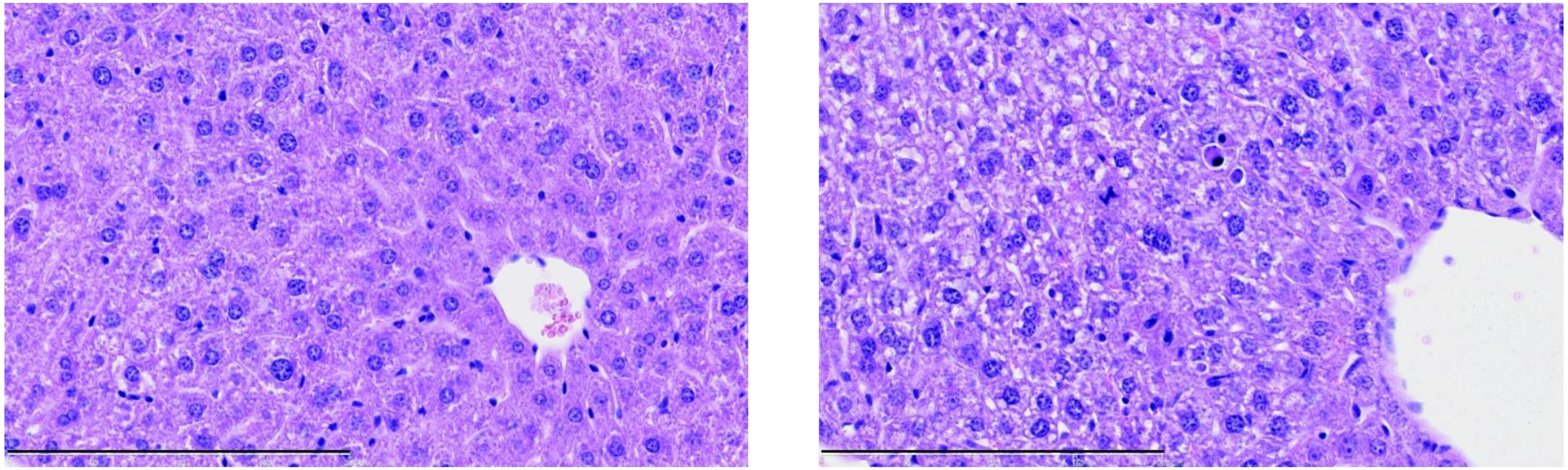

Suppl Fig. S2

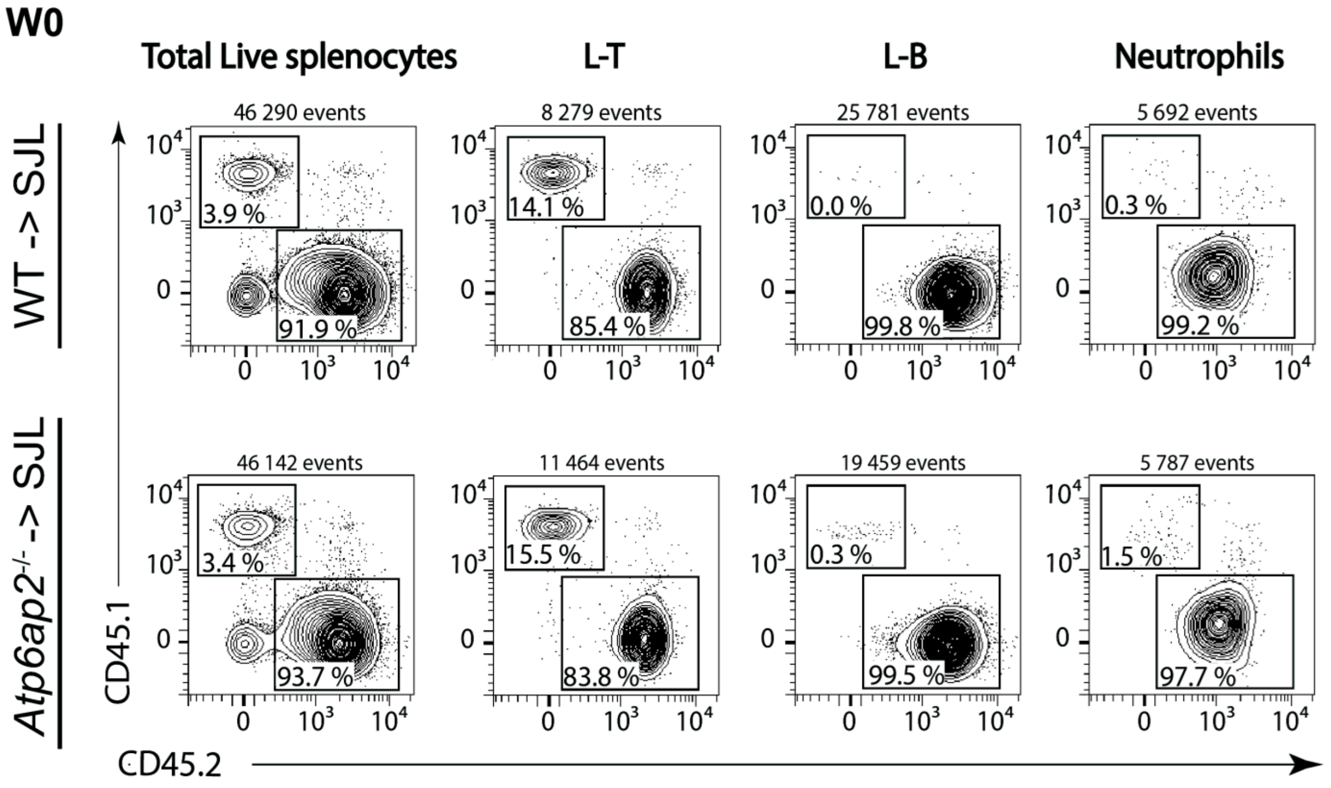
W0
Total Live splenocytes
L-T
L-B
Neutrophils
WT -> SJL
Atp6ap2-/- -> SJL
46 290 events
3.9 %
91.9 %
8 279 events
14.1 %
85.4 %
25 781 events
0.0 %
99.8 %
5 692 events
0.3 %
99.2 %
46 142 events
3.4 %
93.7 %
11 464 events
15.5 %
83.8 %
19 459 events
0.3 %
99.5 %
5 787 events
1.5 %
97.7 %
CD45.1
CD45.2

Suppl Fig S3

A.

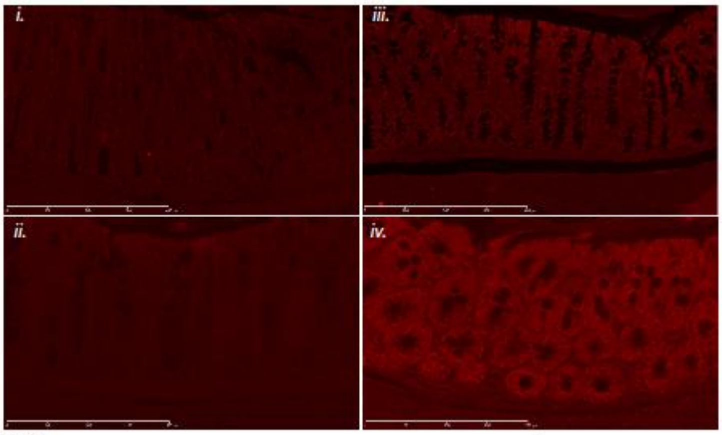

Primary anti-ATP6AP2 - +

B.

anti-ATP6AP2 Ki67

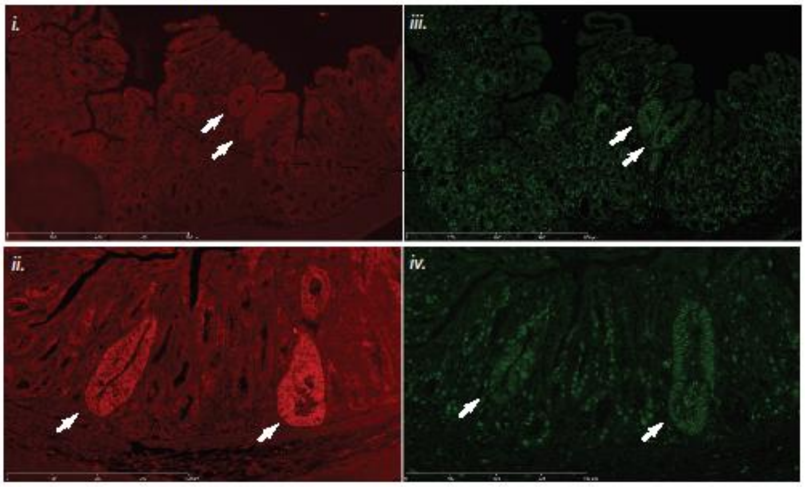

Supplemental Figure S4
